# Assessing the Knowledge, Attitudes, and Practices (KAP) of Sudanese citizens regarding dengue fever prevention and control during the 2025 outbreak in Sudan: a multi-center cross-sectional study

**DOI:** 10.1186/s12889-026-27977-y

**Published:** 2026-05-28

**Authors:** Khabab Abbasher Hussien Mohamed Ahmed, Mohamed Abdallah Mohamed Baraka, Mustafa Mohamed Ibrahim Ali, Mohammed Almurtada Hassan Ali Ahmed, Muotaman Mohammed Abdalla Adam, Rayan Mohammed Abdalla Ahmed, Tho Alyazan Khalil Taher Al-Jabali, Shahd Elzibaer Mohamed Khalil, Mohamed Abdelkarim, Fatima Omer Ibrahim Ahmed, Wadah Ibrahim Rajab Eltoom, Esraa Abdullah Mohamed Baraka, Mustafa Omer Mohammed Mohammed, Elhindy Jalis Younis Makey, Mohammed Izzuldeen Othman Ali, Nazik Elmalaika Obaid Seid Ahmed Husain, Mohamed Elsheikh

**Affiliations:** 1 Faculty of Medicine, University of Khartoum, Khartoum, 11111 Sudan; 2 Faculty of Medicine, University of Science and Technology, Omdurman, 11111 Sudan; 3 Faculty of Medicine, University of Science and Technology, Khartoum, Sudan; 4 University of Science and Technology, Omdurman, Sudan; 5 National Ribat University, Khartoum, Sudan; 6 Faculty of Medicine and Health Sciences, Omdurman Islamic University, Omdurman, Sudan; 7 Faculty of Medicine, ALbyan University, Khartoum, Sudan; 8 Faculty of Medicine, University of Al Fashir, Al Fashir, North Darfur Sudan; 9 Faculty of Medicine, Alfajr College for Sciences and Technology, Khartoum, Sudan; 10 Faculty of Medicine, University of Albutana, RUFA’A, Sudan; 11 Department of Pathology, Faculty of Medicine, University of Tabuk, Tabuk, Saudi Arabia; 12 Department of Physiology, Neuroscience & Behavioral Sciences, School of Medicine, St Georges University, St Georges, Grenada

**Keywords:** Dengue fever, Knowledge, Attitudes, and Practices (KAP), Community awareness, Vector-borne diseases, Sudan

## Abstract

**Background:**

Dengue fever remains a major and expanding public health threat in tropical regions, with outbreaks increasingly affecting fragile and conflict-affected settings. In Sudan, the ongoing armed conflict has compounded vulnerabilities, limited healthcare access, and amplified the importance of community-level prevention and awareness.

**Methods:**

We conducted a community-based, multi-center cross-sectional study to assess knowledge, attitudes, and practices (KAP) related to dengue fever prevention and control during the 2025 outbreak in Sudan. A total of 459 adult participants from seven outbreak-affected states were recruited using an online, convenience-based sampling approach. Due to reliance on online platforms, the sample is predominantly composed of young, educated individuals; this limitation is acknowledged explicitly. Data were collected using a validated KAP questionnaire. Participants were classified as having ‘Good Knowledge’ if they scored ≥ 60% (12/20) on the knowledge section, and ‘Positive Attitude’ if they scored ≥ 60% on the attitude section. Good Practice was defined as a score ≥ 5 out of 9. Data were analyzed using descriptive statistics, chi-square tests, independent t-tests, and binary logistic regression for multivariate analysis. Statistical significance was set at *p* ≤ 0.05.

**Results:**

Overall awareness of the dengue outbreak was high (96.5%). The majority of participants demonstrated good knowledge (96.5%), positive attitudes (99.8%), and good preventive practices (91.3%). However, substantial misconceptions persisted, particularly regarding transmission: 77.3% of respondents incorrectly believed dengue could be spread through contaminated food or water. Knowledge and practice scores were significantly associated with age, gender, and educational level, with older adults, males, and individuals with lower formal education exhibiting poorer outcomes. Attitudes toward dengue prevention were uniformly positive across all demographic groups (*p* > 0.05).

**Conclusion:**

Despite high overall KAP levels, critical knowledge gaps and demographic disparities threaten effective dengue prevention during outbreaks. Targeted, demographic-specific educational interventions addressing misconceptions about transmission and vector behavior are urgently needed to strengthen community-based control efforts, particularly in conflict-affected settings such as Sudan. Future research should prioritize in-person sampling strategies to ensure adequate representation of older adults and individuals with lower levels of formal education.

## Introduction

Dengue fever is a mosquito-borne arboviral infection transmitted by female mosquitoes of the *Aedes* genus (especially *Aedes aegypti* and *A. albopictus*) found mainly in tropical and subtropical regions [1]. Dengue fever is caused by four antigenically different viral serotypes (DENV-1, DENV-2, DENV-3, and DENV-4), all of which can cause severe illness. These are ribonucleic acid (RNA) viruses belonging to the Flaviviridae family, which also includes the yellow fever virus and West Nile virus [1].

Dengue is the most common mosquito-borne disease worldwide [2], with approximately half of the world’s population (~ 4 billion) living in high-risk areas. In recent years, dengue has undergone dramatic global expansion spanning the Americas, Africa, the Middle East, Asia, and the Pacific [3]. The World Health Organization (WHO) reported over 7.6 million dengue cases globally as of 30 April 2024, including 3.4 million confirmed cases, over 16,000 severe cases, and more than 3,000 deaths [4].

Dengue fever presents with a wide spectrum of clinical severity, from asymptomatic infection to potentially fatal forms such as dengue hemorrhagic fever and dengue shock syndrome [5]. Following an incubation period of 5–7 days, illness courses through three phases: febrile, critical, and recovery [6]. The febrile phase lasts 2–7 days, marked by high fever, headache, retro-orbital pain, muscle and joint aches, rash, and minor bleeding manifestations. Warning signs during fever resolution include persistent vomiting, severe abdominal pain, fluid accumulation, mucosal bleeding, hepatomegaly, and rising hematocrit, indicating progression to severe dengue [6, 7]. The critical phase typically lasts 24–48 h and may involve severe plasma leakage leading to shock, respiratory distress, and organ dysfunction. Early recognition and timely supportive care reduce mortality in severe dengue to less than 0.5% [6, 8].

Prevention focuses on avoiding mosquito bites and eliminating *Aedes* breeding sites. Two vaccines are available: Dengvaxia (CYD-TDV) and Qdenga (TAK-003). Dengvaxia requires pre-vaccination serological screening due to safety concerns in seronegative individuals [9, 10]. Qdenga was prequalified by WHO in May 2024 and is approved for children aged 6–16; dengue vaccination must form part of an integrated disease control strategy [11, 12].

Sudan has experienced recurring dengue outbreaks across multiple states [13–16]. A systematic review by Elduma et al. documented dengue cases across 11 of Sudan’s 18 states, with an estimated seroprevalence of approximately 30% [17]. More broadly, dengue has been increasingly recognized as a growing and often underestimated public health threat across the African continent [18]. Globally, the European Centre for Disease Prevention and Control (ECDC) reported more than 4 million dengue cases and over 2,500 fatalities across 101 countries in the first half of 2025 [19].

Sudan’s ongoing armed conflict has severely strained an already fragile health system, disrupted vector control programs, and displaced millions, heightening community vulnerability to infectious disease outbreaks. This study aims to assess community KAP toward dengue fever during the 2025 Sudan outbreak, with the goal of identifying specific knowledge gaps to inform targeted educational and public health interventions.

## Methodology

### Study design and setting

This observational, descriptive, cross-sectional study was conducted to assess the KAP of Sudanese citizens regarding dengue fever prevention and control during the 2025 outbreak. An ethical approval was obtained from the ethical committee of the Local State Ministry of Health in Sudan, and fully informed written consent was obtained from all participants prior to participation. The study was conducted in accordance with the ethical guidelines of the Declaration of Helsinki. Convenience sampling was used via online platforms (social media-based survey), targeting participants from seven outbreak-affected states: Khartoum, Red Sea, Al Qadarif, Gezira, North Darfur, Kassala, and River Nile. The questionnaire was distributed via Google Forms through Facebook, LinkedIn, and Telegram. Data collection was conducted in August 2025 over two weeks. It is explicitly acknowledged that this online convenience sampling approach introduces selection bias, systematically favoring younger, educated, and internet-connected individuals, and this limitation should be considered when interpreting all findings.

### Study participants and sample size

The study included adult Sudanese citizens (≥ 18 years) with access to stable internet connections. Using a 95% confidence level, expected prevalence of 50%, and margin of error of 5%, the initial sample size was calculated as 385. A 19% non-response allowance yielded a final target of 459 participants.

### Data collection tools and scoring criteria

A validated questionnaire previously used in Yemen [20] was adapted for Sudanese context after piloting. The questionnaire comprised four sections addressing sociodemographics, knowledge (20 items), attitudes (6 items), and practices (9 items). Participants scoring ≥ 12 out of 20 (≥ 60%) on knowledge were classified as having ‘Good Knowledge’; those scoring ≥ 18 out of 30 (≥ 60%) on attitudes were classified as having ‘Positive Attitude’; and those scoring ≥ 5 out of 9 on practices were classified as having ‘Good Practice’. These thresholds were adapted from the original validated instrument [20].

### Statistical analysis

Data were analyzed using SPSS version 25. Frequencies and percentages were used for categorical variables; means ± SD for continuous variables. Chi-square tests evaluated associations between categorical variables and KAP classifications. Independent t-tests compared mean scores between binary groups. Binary logistic regression was performed for variables significant in univariate analysis to identify independent predictors of poor knowledge and poor practice. Statistical significance was set at *p* ≤ 0.05 with 95% CI.

## Results

### Participant characteristics

**Fig. 1:**
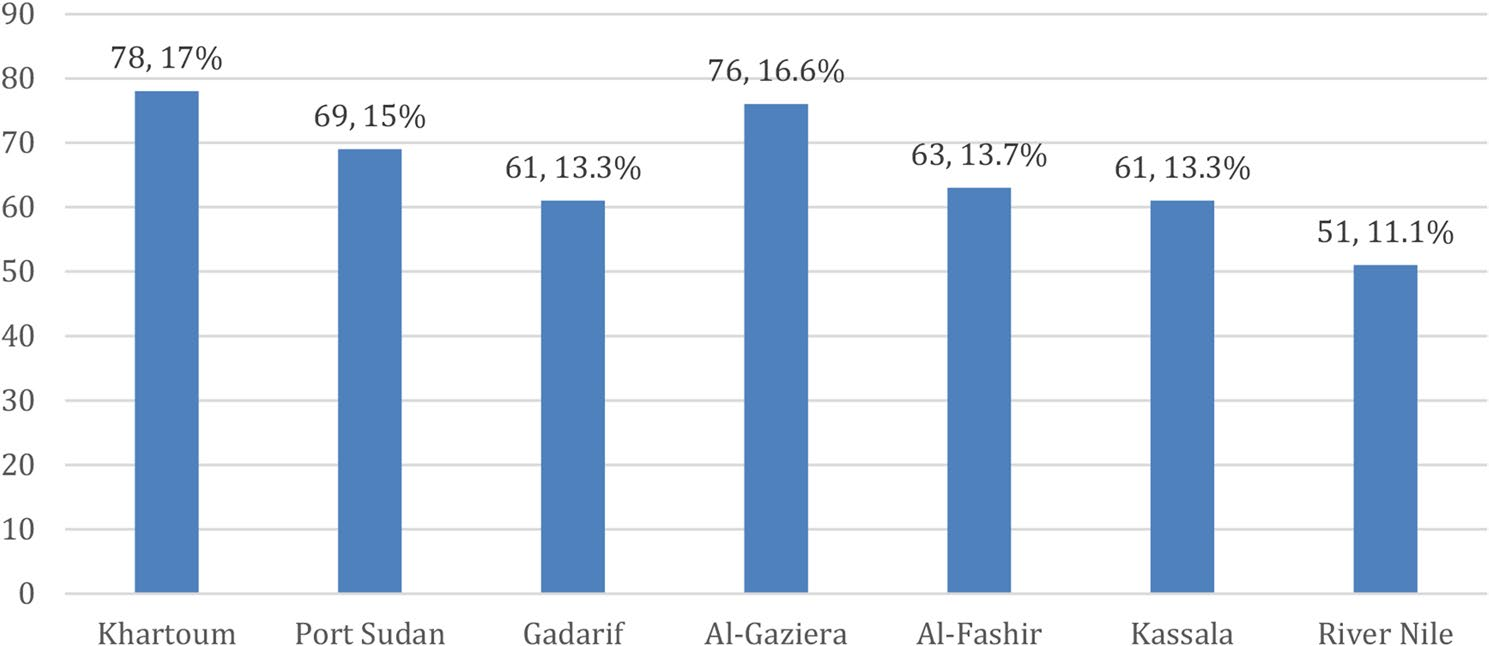
Distribution of dengue fever study participants by residence, Sudan. *N* = 459

This study included 459 participants. The sample was predominantly young (84.1%, aged 18–35), approximately balanced by sex (48.4% male, 51.6% female), and highly educated (71.9% university; 11.8% postgraduate) (Table 1). The geographical distribution of participants across the seven affected states is illustrated in Fig. 1.

**Table 1:** Demographic data of study participants, Sudan. *N* = 459

Demographic Parameter		Frequency(%)
Age	18–35	386 (84.1%)
	36–50	49 (10.7%)
	51–65	19 (4.1%)
	66–80	5 (1.1%)
Gender	Males	222 (48.4%)
	Females	237 (51.6%)
Educational Level	Illiterate	7 (1.5%)
	Elementary School	19 (4.1%)
	Secondary School	49 (10.7%)
	University	330 (71.9%)
	Postgraduate Level	54 (11.8%)

### Knowledge scores and misconceptions

**Fig. 2:**
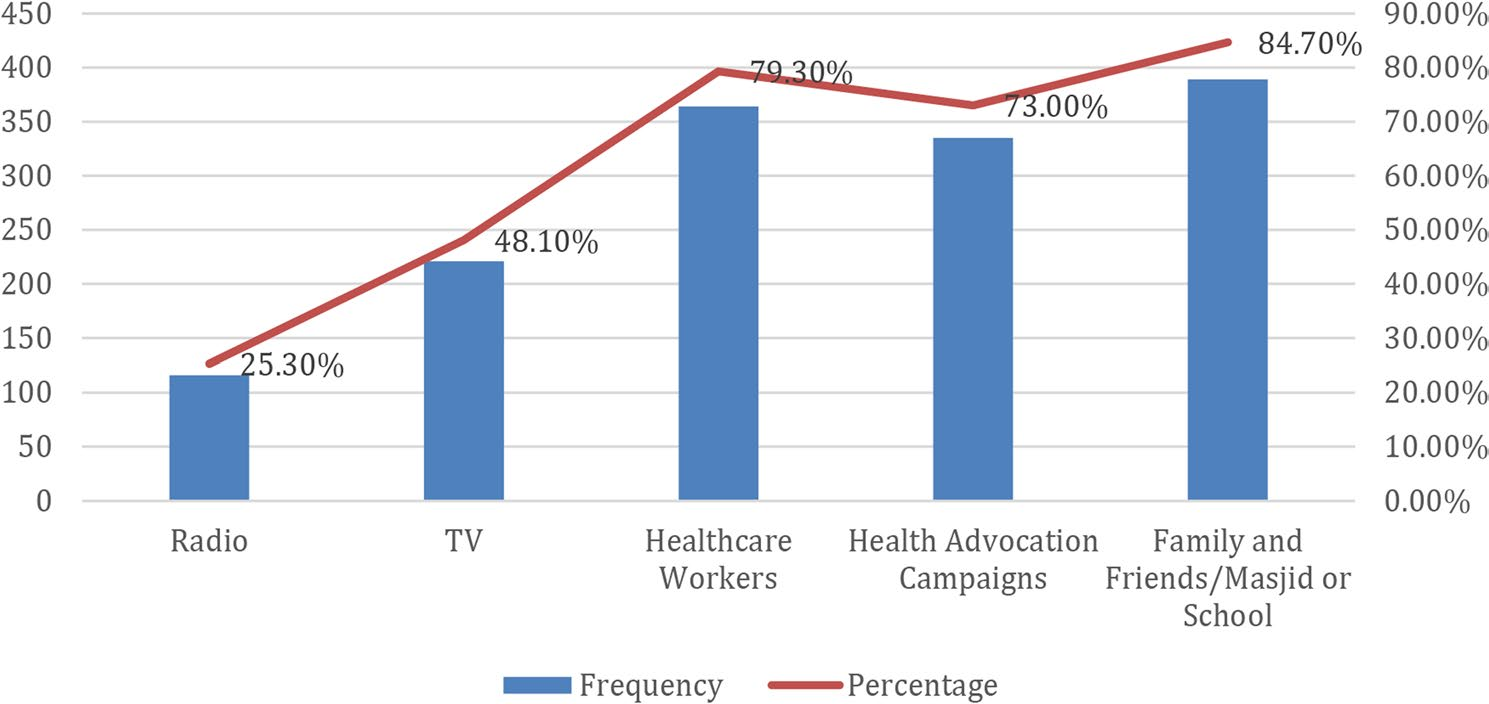
Sources of information about dengue fever among study participants, Sudan. *N* = 459

Participants demonstrated high overall knowledge, with a mean score of 15.5 ± 2.4 and 96.5% (*n* = 443) classified as having good knowledge (Fig. 2). Recognition of primary symptoms was high: fever (97.6%), headache (95.6%), and joint pain (91.5%). However, awareness of the dengue rash (51.2%), eye pain (63.0%), and bleeding as a severe manifestation (recognized by 70.8%) was substantially lower (Table 2).

**Table 2:** Knowledge assessment parameters among study participants, Sudan. *N* = 459

Knowledge Parameters	Incorrect (%)	Correct (%)
Symptoms of Dengue Fever		
Fever	11 (2.4%)	448 (97.6%)
Headache	20 (4.4%)	439 (95.6%)
Joint Pain	39 (8.5%)	420 (91.5%)
Muscle Pain	68 (14.8%)	391 (85.2%)
Eye Pain	170 (37%)	289 (63%)
Skin Rash	224 (48.8%)	235 (51.2%)
Bleeding	134 (29.2%)	325 (70.8%)
Transmission of the Dengue Fever		
All Mosquitoes transmit Dengue Fever	124 (27%)	335 (73%)
Aedes Aegypti Transmits Dengue Fever	106 (23.1%)	353 (76.9%)
Flies Do Not Transmit Dengue Fever	178 (38.8%)	281 (61.2%)
Direct Contact with Patients Does Not Transmit Dengue Fever	177 (38.6%)	282 (61.4%)
Contaminated Food and Drinks Transmit Dengue Fever	355 (77.3%)	104 (22.7%)
Stagnant Water Increases Vector Spread	29 (6.3%)	430 (93.7%)
Uncovered Water containers Increase Vector Spread	46 (10%)	413 (90%)
Time that the Vector Likes to Feed	301 (65.6%)	158 (34.4%)
Vector Control Data		
Insecticides Use is a Part of Vector Control	11 (2.4%)	448 (97.6%)
Tightly Covering Water Containers is a Part of Vector Control	17 (3.7%)	442 (96.3%)
Drying Stagnant Water is a Part of Vector Control	17 (3.7%)	442 (96.3%)
Using Mosquito Nets and Insect Nettings is a Part of Vector Control	4 (0.9%)	455 (99.1%)
Using Mosquito Repellent is a Preventive Measure	30 (6.5%)	429 (93.5%)
Mean Knowledge Score	**(Mean ± SD)**	**(15.5 ± 2.4)**

Significant transmission misconceptions were identified: 77.3% (*n* = 355) incorrectly attributed dengue to contaminated food or water. Only 34.4% correctly identified daytime feeding behavior of *Aedes aegypti*. Knowledge of vector control measures was high (> 90%) (Table 2).

### Attitudes toward dengue fever

**Fig. 3:**
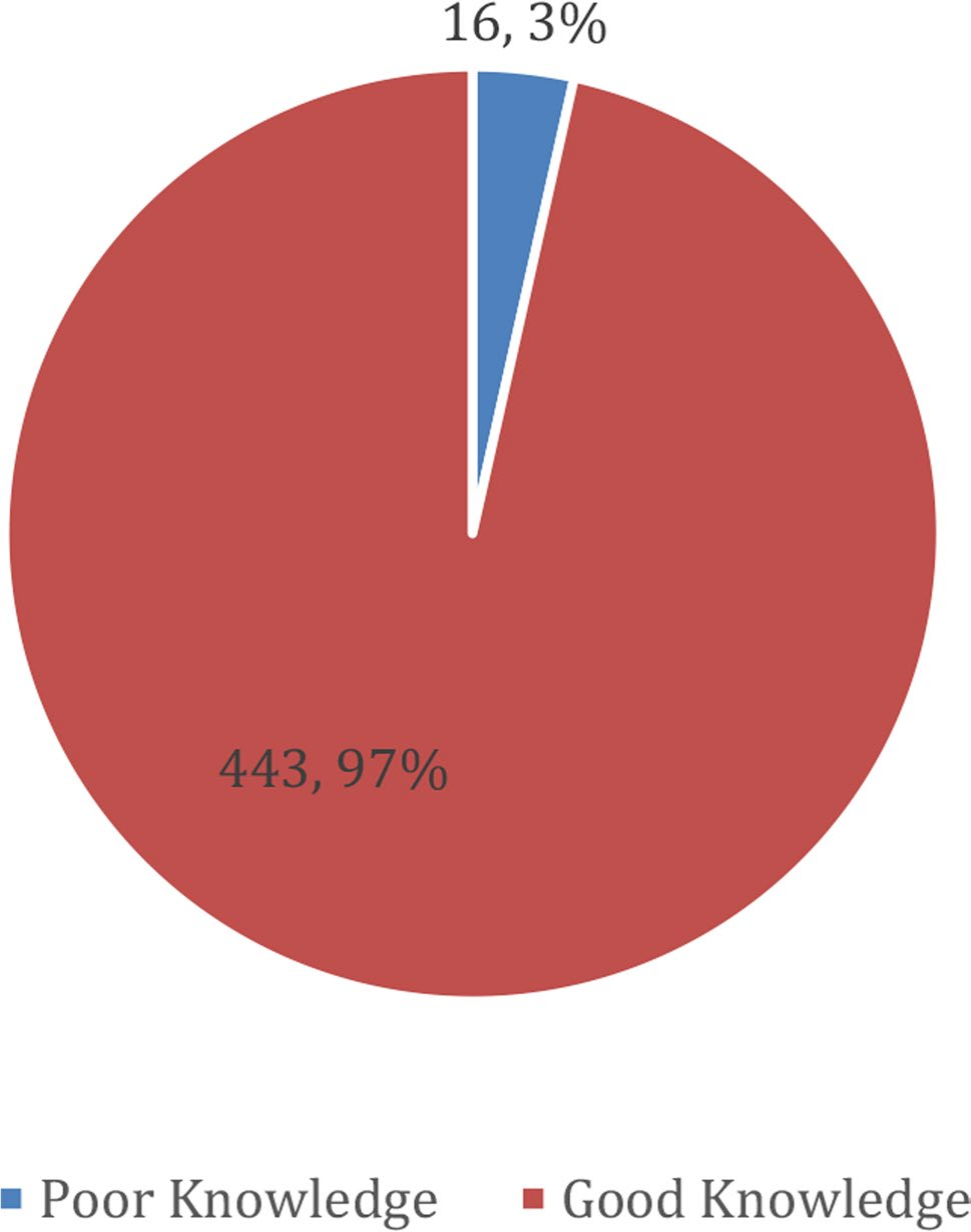
Assessment of knowledge of dengue fever among study participants, Sudan. *N* = 459

The mean attitude score was 26.0 ± 2.9, with 99.8% (*n* = 458) classified as having a good attitude (Fig. 3). A large majority recognized dengue as preventable (92.4%), infectious (74.7%), and dangerous (56.9%), with 93.1% reporting personal perceived risk. Over 95.5% supported community participation in vector control. Attitude classification was not significantly associated with age, sex, or educational level (*p* > 0.05 for all comparisons). Specific p-values are reported in Table 3. Demographic associations with attitude classification are presented in Table 4.

**Table 3:** Attitude assessment parameters among study participants, Sudan. *N* = 459

Attitude Parameters	Strongly Disagree	Disagree	Neutral	Agree	Strongly Agree
Dengue Attitude Elements	1 (0.2%)	7 (1.5%)	13 (2.8%)	143 (31.2%)	295 (64.3%)
Dengue Fever is a Dangerous Disease		102 (22.2%)	96 (20.9%)	122 (26.6%)	139 (30.3%)
Dengue Fever is an Infectious Disease		47 (10.2%)	69 (15%)	153 (33.3%)	190 (41.4%)
I am Exposed to Contracting the Disease		8 (1.7%)	24 (5.2%)	171 (37.3%)	256 (55.8%)
Dengue Fever is a Preventable Disease		11 (2.4%)	24 (5.2%)	141 (30.7%)	283 (61.7%)
Stagnant Waters in Hollowed Tires and Open Water Bottles is a Good Habitat for Vector Reproduction		1 (0.2%)	6 (1.3%)	107 (23.3%)	345 (75.2%)
Community Should Actively Participate in Vector Control		7 (1.5%)	13 (2.8%)	143 (31.2%)	295 (64.3%)
Dengue Attitude Score		**(Mean ±**	**SD)**	**(26 ± 2.9)**	

**Table 4:** Association between Attitude and Demographics Among Study Participants, Sudan. *N* = 459

Parameters	Poor Attitude	Good Attitude	Total	*P* value
Age Group				0.979
18–35	1 (0.3%)	385 (99.7%)	386 (100%)	
36–50	0 (0.0%)	49 (100.0%)	49 (100%)	
51–65	0 (0.0%)	19 (100.0%)	19 (100%)	
66–80	0 (0.0%)	5 (100.0%)	5 (100%)	
Gender				0.301
Males	1 (0.5%)	221 (99.5%)	222 (100%)	
Females	0 (0.0%)	237 (100.0%)	237 (100%)	
Educational Level				< 0.001***
Illiterate	0 (0.0%)	7 (100.0%)	7 (100%)	
Elementary School	1 (5.3%)	18 (94.7%)	19 (100%)	
Secondary School	0 (0.0%)	49 (100.0%)	49 (100%)	
University	0 (0.0%)	330 (100.0%)	330 (100%)	
Postgraduate Level	0 (0.0%)	54 (100.0%)	54 (100%)	
Total	1 (0.2%)	458 (99.8%)	459 (100%)	

### Preventive practices

**Fig. 4:**
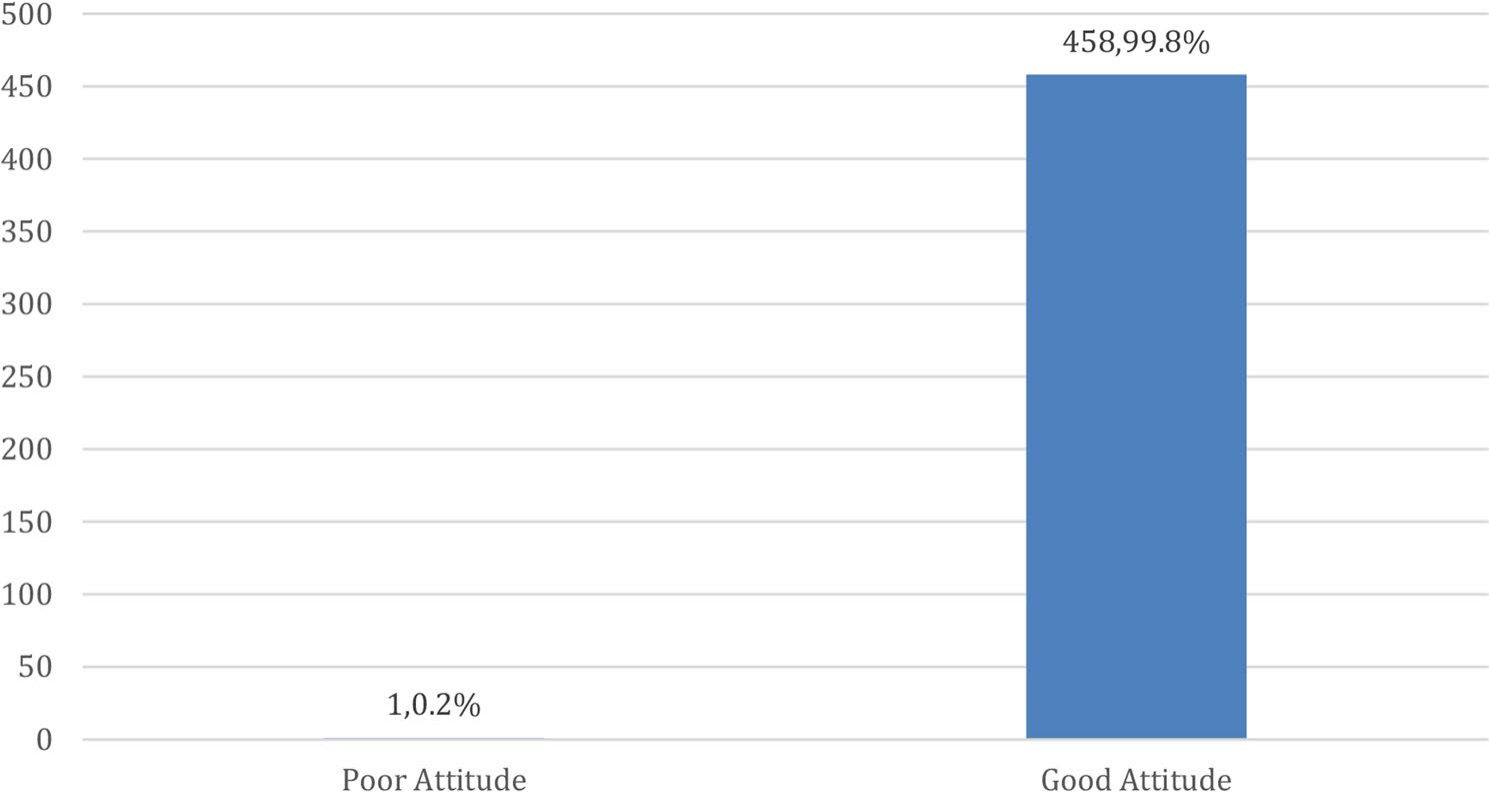
Assessment of attitude regarding dengue fever among study participants, Sudan. *N* = 459

**Fig. 5:**
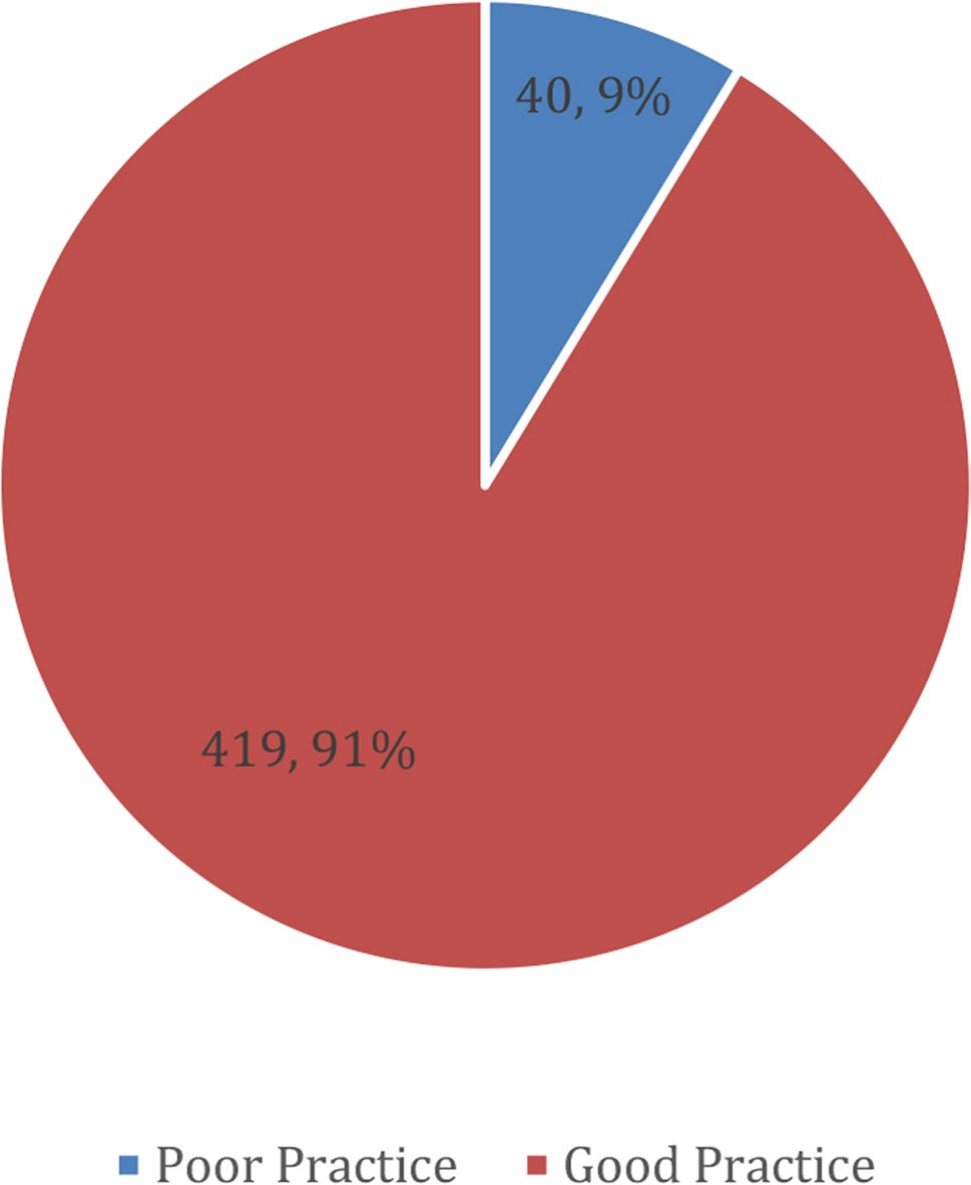
Assessment of practices regarding dengue fever among study participants, Sudan. *N* = 459

The mean practice score was 7.9 ± 2.1, with 91.3% (*n* = 419) classified as having good practice (Figs. 4 and 5). High proportions reported using insecticides (87.8%), sleeping under mosquito nets (91.7%), using fans (85.2%) or repellents (86.7%), disposing of breeding sites (87.1%), and covering water containers (92.2%) (Table 5).

**Table 5:** Practice parameters among study participants, Sudan. *N* = 459

Mean Practice Score	(Mean ± SD)	(7.9 ± 2.1)
Practice Parameters	No	Yes
I Used insecticides to limit vector spread	56 (12.2%)	403 (87.8%)
Mosquito Nets Are Available to Me	31 (6.8%)	428 (93.2%)
I Sleep Under Mosquito Nets	38 (8.3%)	421 (91.7%)
I Use Fans to Repel Mosquitos	68 (14.8%)	391 (85.2%)
I Use Insects Netting to Decrease Mosquito Contact	78 (17%)	381 (83%)
I Dispose Opened Containers and Hollow Tires and Uncovered Water Bottles	59 (12.9%)	400 (87.1%)
I Used Mosquito Repellant	61 (13.3%)	398 (86.7%)
I Cover My Body with Thick and Long Clothes	88 (19.2%)	371 (80.8%)
I Cover Water Containers in the House	36 (7.8%)	423 (92.2%)

### Factors associated with knowledge and practice

Knowledge classification was significantly associated with age (*p* = 0.002), sex (*p* = 0.007), and educational level (*p* < 0.001). Poor knowledge was disproportionately observed in older age groups (20.0% in the 66–80 group), males (5.9% vs. 1.3% in females), and those with lower education (28.6% illiterate; 26.3% elementary-level) (Table 6). Chi-square statistics and odds ratios from logistic regression are presented in Tables 6 and 7.

**Table 6:** Association between knowledge and demographics among study participants, Sudan. *N* = 459

Parameters	Poor Knowledge	Good Knowledge	Total	*P* value
Age Group				0.002*
18–35	12 (3.1%)	374 (96.9%)	386 (100%)	
36–50	0 (0.0%)	49 (100%)	49 (100%)	
51–65	3 (15.8%)	16 (84.2%)	19 (100%)	
66–80	1 (20.0%)	4 (80.0%)	5 (100%)	
Gender				0.007*
Males	13 (5.9%)	209 (94.1%)	222 (100%)	
Females	3 (1.3%)	234 (98.7%)	237 (100%)	
Educational Level				< 0.001***
Illiterate	2 (28.6%)	5 (71.4%)	7 (100%)	
Elementary School	5 (26.3%)	14 (73.7%)	19 (100%)	
Secondary School	4 (8.2%)	45 (91.8%)	49 (100%)	
University	5 (1.5%)	325 (98.5%)	330 (100%)	
Postgraduate Level	0 (0.0%)	54 (100.0%)	54 (100%)	
Total	16 (3.5%)	443 (96.5%)	459 (100%)	

**Table 7:** Association between practice and demographics among study participants, Sudan. *N* = 459

Parameters	Poor Practice	Good Practice	Total	*P* value
Age Group				0.014*
18–35	29 (7.5%)	357 (92.5%)	386 (100%)	
36–50	5 (10.2%)	44 (89.8%)	49 (100%)	
51–65	4 (21.1%)	15 (78.9%)	19 (100%)	
66–80	2 (40.0%)	3 (60.0%)	5 (100%)	
Gender				< 0.001***
Males	31 (14.0%)	191 (86.0%)	222 (100%)	
Females	9 (3.8%)	228 (96.2%)	237 (100%)	
Educational Level				< 0.001***
Illiterate	2 (28.6%)	5 (71.4%)	7 (100%)	
Elementary School	6 (31.6%)	13 (68.4%)	19 (100%)	
Secondary School	7 (14.3%)	42 (85.7%)	49 (100%)	
University	22 (6.7%)	308 (93.3%)	330 (100%)	
Postgraduate Level	3 (5.6%)	51 (94.4%)	54 (100%)	
Total	40 (8.7%)	419 (91.3%)	459 (100%)	

Practice classification was significantly associated with age (*p* = 0.014), sex (*p* < 0.001), and educational level (*p* < 0.001). Poor practice increased progressively with age (7.5% in 18–35 to 40.0% in 66–80 group). Males reported poor practice at nearly four times the rate of females (14.0% vs. 3.8%). Participants with only elementary education had 31.6% poor practice, approximately five times higher than university-educated participants (6.7%) (Table 7).

## Discussion

The present study assessed KAP toward dengue fever among Sudanese citizens during the 2025 outbreak. Most participants demonstrated good knowledge, positive attitudes, and good preventive practices; however, critical misconceptions and demographic disparities warrant targeted intervention.

### Knowledge

The high proportion of participants with good knowledge (96.5%) substantially exceeds prior estimates from Sudanese medical students (57.9%) [21] and Yemeni community members (53.5%) [20]. This elevated performance may reflect heightened personal motivation to seek information during an active, locally experienced outbreak. The endemic co-presence of malaria, which shares preventive measures with dengue, may also contribute to informational crossover. The high educational level of this convenience sample is also likely to have inflated aggregate knowledge estimates, and findings should not be generalized to less-educated subgroups.

Recognition of cardinal symptoms—fever, headache, joint pain—was high and consistent with prior studies [20–22]. The notably lower recognition of the dengue rash (51.2%), compared to 90% reported by Ahmed et al. [21], may reflect the displacement of less-prominent clinical features in community awareness by more acutely alarming symptoms during an ongoing outbreak.

The most concerning knowledge gap involved transmission: 77.3% attributed dengue to contaminated food or water, higher than in Yemen (~ 52%) [20]. This misconception may reflect cognitive consolidation of preventive knowledge across communicable diseases, as well as inadequate specificity in health education materials. Misdirected preventive beliefs of this nature risk diverting individual and community efforts away from effective vector control measures.

Only 34.4% correctly identified daytime feeding of *Aedes aegypti*, lower than Yemen (56%) [20], Sudanese medical students (66%) [21], and Malaysia (91.8%) [22]. This gap is clinically significant, as communities habituated to nocturnal malaria vector precautions may remain insufficiently protected during daytime hours when dengue transmission predominantly occurs.

### Attitudes

Near-universal positive attitudes (99.8%) substantially exceed Yemen (64%) [20] and Malaysia (~ 47%) [22]. This may reflect heightened threat salience under active outbreak conditions in a conflict-affected setting where disease burden is immediately tangible. The absence of significant demographic variation in attitudes (*p* > 0.05) indicates that favorable attitudes are broadly shared, and that the primary barriers to effective prevention in this population are specific knowledge deficits and practice gaps rather than motivational deficits.

### Practices

High rates of good practice (91.3%) likely reflect the co-endemicity of malaria, which has established household-level vector control behaviors [23–25]. However, the long-standing emphasis on nocturnal mosquito protection for malaria prevention may leave communities insufficiently vigilant against the daytime-biting *Aedes* vector responsible for dengue. Sustained behavioral interventions addressing this specific gap are warranted, as high knowledge does not automatically translate to appropriate practice [26].

### Demographic disparities

The convergence of knowledge and practice deficits in older adults, males, and individuals with lower formal education represents the most actionable finding of this study. The association between educational level and both knowledge and practice is consistent with findings from Colombia [27] and reinforces that aggregate performance does not reflect the vulnerability of less-educated subgroups. Poorer knowledge in older adults is of particular concern given associations with increased risk of severe dengue outcomes [28]. The disproportionate engagement of females in household vector control practices [29, 30] underscores the need for gender-sensitive interventions targeting males.

### Limitations

Online convenience sampling introduces substantial selection bias, resulting in a sample disproportionately composed of young, educated, digitally connected individuals. Findings cannot be generalized to older, less-educated, or rural populations. The ongoing armed conflict precluded proportional sampling across strata. The cross-sectional design precludes causal inference, and self-reported practices are subject to social desirability bias. Adaptation of the Yemeni questionnaire may not fully capture Sudan-specific knowledge constructs.

### Recommendations

1. Develop targeted educational interventions for older adults, males, and individuals with lower formal education using visual and oral communication formats (e.g., radio, community health workers, illustrated pamphlets).
2. Address the misconception of food/water-borne dengue transmission in all public health messaging, emphasizing vector-borne transmission and *Aedes* daytime feeding behavior.
3. Leverage existing malaria-prevention infrastructure to develop comparative educational materials differentiating dengue and malaria prevention, reinforcing daytime protection practices.
4. Include warning signs of severe dengue in public campaigns to promote early care-seeking.
5. Future research should employ in-person, community-based sampling to obtain representative data from the most vulnerable subgroups.

## Conclusion

Despite high overall KAP levels, this study identified critical misconceptions regarding dengue transmission and demographic disparities in knowledge and practice that represent meaningful obstacles to community-based dengue control in Sudan. Older adults, males, and individuals with lower formal education constitute priority subgroups requiring targeted, culturally adapted, and literacy-appropriate interventions. Strengthening dengue-specific health education in conflict-affected settings is urgently needed to avert preventable morbidity.

## Data Availability

The datasets used and analysed during the current study are available from the corresponding author upon reasonable request.
